# Downregulation of Notch Signaling Pathway in Late Preterm and Term Placentas from Pregnancies Complicated by Preeclampsia

**DOI:** 10.1371/journal.pone.0126163

**Published:** 2015-05-11

**Authors:** Persefoni Fragkiadaki, Nikolaos Soulitzis, Stavros Sifakis, Demetrios Koutroulakis, Victor Gourvas, Nikolaos Vrachnis, Demetrios A. Spandidos

**Affiliations:** 1 Laboratory of Clinical Virology, Medical School, University of Crete, Heraklion, Crete, Greece; 2 Department of Obstetrics & Gynecology, University Hospital of Heraklion, Crete, Greece; 3 2^nd^ Department of Obstetrics & Gynaecology, Aretaieion Hospital, National and Kapodistrian University of Athens, Athens, Greece; East Carolina University, UNITED STATES

## Abstract

Preeclampsia (PE) is a major cause of maternal mortality and morbidity, affecting 3–5% of all pregnancies. The Notch signaling pathway plays an important role during placental development, activating several target genes. Defects in the Notch pathway have adverse effect on placentation. The aim of this study was to investigate the expression of receptors *NOTCH1*,*-2*,*-3*,*-4*, ligands *DLL1*,*-3*,*-4*, *JAG1*,*-2* and target genes *HEY1*,*-2* in placental tissue samples from 20 late preterm or term pregnancies complicated by PE versus 20 normal pregnancies. mRNA levels of the studied molecules were measured by quantitative Real-Time PCR (qRT-PCR), while the protein expression of the intracellular domain of NOTCH2 (NICD2) and NOTCH3 (NICD3) was measured by Western Blot (WB). qRT-PCR analysis revealed that *NOTCH1*, *NOTCH4* and *DLL1* were not expressed in the placenta. On the contrary, *NOTCH2*, *NOTCH3*, *DLL3*, *DLL4*, *JAG1*, *JAG2*, *HEY1* and *HEY2* mRNA levels were downregulated in PE samples vs. controls (p<0.01). WB confirmed that NICD2 (p = 0.014) and NICD3 (p<0.001) protein levels were also lower in PE specimens. Statistical analysis revealed several significant associations: of *NOTCH3* mRNA expression with smoking during pregnancy (p = 0.029), of NICD3 protein levels (p = 0.028) and *DLL3* mRNA levels (p = 0.041) with birth weight centile, and of *HEY2* transcript levels with parity (p = 0.034) and mode of delivery (p = 0.028). Our results suggest that Notch pathway downregulation is associated with PE. Further studies are required in order to determine the role of these molecules in PE pathogenesis and to evaluate their potential use for the early detection and treatment of PE.

## Introduction

Preeclampsia (PE) affects 3–5% of all pregnancies worldwide and is a major cause of maternal mortality and morbidity [1]. PE is a consequence of diverse pathological processes involving impaired implantation, endothelial dysfunction and systemic inflammation, and it is characterized by hypertension and proteinuria during pregnancy. Chronic renal disease, obesity and diabetes are among several risk factors associated with PE [2].

The Notch signaling pathway is an evolutionarily conserved intercellular signaling mechanism, which is involved in cell fate decisions and pattern formation during development [3]. NOTCH1-4 receptors are single-pass transmembrane proteins that are activated by Delta (DLL1,-3,-4) and Jagged/Serrate (JAG1,-2) ligands [4]. Each of these proteins shows cell-type- and tissue-specific expression during development. This ligand-receptor interaction leads to the proteolytic cleavage and release of the NOTCH intracellular domain (NICD), which translocates to the nucleus, where it interacts with the DNA with the help of transcription factor RBPJK, inducing the expression of numerous target genes [5].

Members of the Notch signaling pathway have been detected in the developing placenta, where they play an important role in placentation [6]. In mice, Notch signaling controls fetal angiogenesis, maternal circulatory system development and spongiotrophoblast development [7]. In the human term placenta, NOTCH2 and JAG2 proteins are involved in the regulation of trophoblast fate decisions, vasculogenesis and/or feto-maternal trafficking [6], while NOTCH2 also regulates maternal blood sinus formation. Abnormal expression of the Notch ligand *JAG1* results in the failure of endovascular remodeling [8], whereas *Notch2* deletion in mice leads to a marked reduction in the placental perfusion and arterial invasion by cytotrophoblast cells (CTBs) [7]. All of the above suggest a possible connection between the Notch signaling pathway and PE.

NICD activates the transcription of several target genes, most notably the *HEY1* and *HEY2* genes, which are required for vascular development [9]. These genes are expressed in the developing cardiovascular system, including the heart, endothelial cells and vascular smooth muscles [3, 10], as well as in the placental labyrinth, while in mice *Hey1* is additionally expressed in some trophoblast cells in the ectoplacental cone [7]. Knockout of any of these genes leads to placental vascular deficiencies and to early embryonic lethality, due to major vascular and cardiac defects [9].

The expression of Notch signaling pathway molecules is localized and not universal across the placenta. JAG1, DLL1 and DLL4 are mainly localized in capillary endothelial cells in tertiary villi, while JAG1 is additionally detected in large vessels and perivascular cells [6]. NOTCH4 is also predominantly expressed in vascular endothelial cells [11]. On the contrary, NOTCH2 protein is expressed weakly and sporadically in CTB progenitor cells, whereas NOTCH3 protein expression is present at all stages of CTB differentiation [8].

Because there are conflicting reports regarding the expression of Notch signaling pathway members in the placenta [12], the purpose of this study was to measure the expression of *NOTCH* (1–4) receptors, its ligands *DLL* (1, 3, 4) and *JAG* (1, 2) and its target genes *HEY* (1, 2), in human late preterm and term placentas from normal and preeclamptic pregnancies, in order to identify potential differences that could clarify their role in this pregnancy complication.

## Materials and Methods

### Placental collection and processing

Placentas were obtained immediately after delivery (vaginal delivery or elective caesarean section) from 20 women with normal pregnancies (gestation period 37–40 wks) and from 20 women with pregnancies complicated by PE (gestation period 34–40 wks, with 7/20 pregnancies <37wks). Six basal plate biopsy specimens of the medial part of the maternal-fetal interface were obtained from each placenta in such a way that each sample contained the decidua basalis and villous placenta, but not the chorionic plate, in order to avoid sample contamination from maternal tissues. Areas involving gross calcifications or infarcts were avoided. Contamination from fetal membranes was also minimized. Tissue biopsies were snap-frozen in liquid nitrogen and stored at -80°C. Three of the above six biopsy specimens were randomly selected for our measurements, while the other three were sent, as is indicative in PE pregnancies, for routine histological examination, which was consistent with the underlying pathology. The Ethics Committee of the University Hospital of Heraklion approved the study, and written informed consent was obtained from all participants.

### Clinical Definitions and Sample Description

Preeclampsia was defined as hypertension in previously normotensive women after 20 weeks of gestation (systolic blood pressure of ≥140 mmHg or diastolic blood pressure of ≥90 mmHg) on at least two occasions, associated with proteinuria (≥300 mg in a 24-hour urine collection or one dipstick measurement of ≥2+) [13]. Control group included women with uncomplicated, normotensive singleton pregnancies who delivered healthy, appropriate-for-gestational-age babies. Exclusion criteria were stillbirth, multiple gestations, chorioamnionitis, pre-pregnancy hypertension, renal disease, as well as chromosomal abnormalities and fetal anatomical defects.

### RNA extraction and cDNA preparation

Tissues (~100mg) were homogenized with a Mortar and Pestle with the help of liquid nitrogen. RNA was extracted using TRIzol reagent (Invitrogen, Carlsbad, CA), followed by chloroform addition and centrifugation. Total RNA was precipitated from the supernatant with isopropanol, washed with 75% ethanol and resuspended in 30μl of diethylpyrocarbonate (DEPC)-treated water. RNA concentration and purity were calculated after measuring on a Nanodrop 1000 spectrophotometer (NanoDrop Products, Wilmington, DE) its 260 nm absorbance and 260/280 nm absorbance ratio, respectively.

cDNA was synthesized using the PrimerScript RT reagent kit (Takara Bio, Otsu, Japan). In detail, 1μg of total RNA, 2μl of random hexamers and 1μl dNTPs were heated at 65°C for 5min, in order to remove RNA secondary structures, and placed on ice until the addition of cDNA synthesis mix, which contained 5× PrimerScript Buffer, 20 units RNase Inhibitor and 200 units PrimerScript RTase. The final mix (volume 20μl) was incubated for 10min at 30°C for primer extension, and cDNA synthesis was conducted at 50°C for 50min. The reaction was terminated by heating at 95°C for 5min. cDNA was stored at -20°C until use.

### Quantitative Real-Time Polymerase Chain Reaction (qRT-PCR) assay

*NOTCH1*, *NOTCH2*, *NOTCH3*, *NOTCH4*, *DLL1*, *DLL3*, *DLL4*, *JAG1*, *JAG2*, *HEY1* and *HEY2* mRNA expression was measured using a qRT-PCR assay with the SYBR Green I dye. The housekeeping gene, beta-Actin (ACTB), was used as an internal control, in order to normalize the studied genes’ expression levels. The mRNA-specific primers, which were designed with the Lasergene software (DNASTAR, Madison, WI) and span at least one intron with an average length >800 bp, are listed in Table 1. Their specificity was verified with the BLAST program (http://www.ncbi.nlm.nih.gov/BLAST). cDNA (1μl) from PE or control samples was amplified in a PCR reaction containing 2× Maxima SYBR Green qPCR Master Mix (Fermentas, Vilnius, Lithuania) and 300nM of each primer in a final volume of 20μl. To ensure the accuracy of the quantification measurements, a representative pool of all the samples was diluted in a series of six 2× dilutions and was run on the same plate, in order to construct a standard curve for the quantification process. After initial denaturation at 95°C for 10min, samples were subjected to 40 cycles of amplification, comprised of denaturation at 95°C for 20s, annealing at 60°C for 30s and elongation at 72°C for 30s, followed by a melt curve analysis in which the temperature was increased from 60°C to 95°C at a linear rate of 0.2°C/sec. Data collection was performed during both annealing and extension, with two measurements at each step, and at all times during the melt curve analysis. PCR experiments were conducted on an Mx3000P real-time PCR thermal cycler using software version 4.01 (Stratagene, La Jolla, CA). After amplification, standard curves were constructed from the samples used in the series of consecutive dilutions. Subsequently, using these standard curves and the Ct value of the samples, we calculated the mRNA expression of the genes studied. Samples with no amplification plots or with dissociation curves that exhibited signs of primer-dimer formation or by-products were excluded. To normalize the mRNA expression of each gene, its value was divided by the β-Actin mRNA value. The normalized values of PE samples were divided by the average normalized values of normal samples. The result of this division provided the relative expression of PE specimen in relation to the control group. This mathematical process is summarized in the following formula:

$$Normalized\frac{Sample}{Control}=\frac{{\left(1+E_{gene}\right)}^{-\Delta Ct_{gene}}}{{\left(1+E_{ACTB}\right)}^{-\Delta Ct_{ACTB}}}$$

**Table 1:** Primer sequences, annealing temperatures and amplicon sizes for the qRT-PCR analysis of the studied genes.

Primer pair	Sequence (5’– 3’)	Annealing temperature (°C)	Amplicon size (bp)
***NOTCH1***	CAG CCT CAA CAT CCC CTA CAA G	60	127
	GCA GCC CAC GAA GAA CAG AA		
***NOTCH2***	GTG GAT GGG GTC AAC ACT TAC A	60	169
	CAC TCC AGC CGT TGA CAC ATA C		
***NOTCH3***	TGG CGC CTC TTC AAC AAC A	60	189
	ATC CCA GCC GCA CTC CTC		
***NOTCH4***	TGC CTC TGC CCC TCT GGT	60	106
	TGG CCT TGT CTT TCT GGT CCT		
***DLL1***	GGG AGC GTG GGG AGA AAG T	60	175
	CAG CCT GGA TAG CGG ATA CAC T		
***DLL3***	GCC CGT CCT CTG CTA CCA C	60	160
	GTC ACC CCG CTC ACC TCA C		
***DLL4***	TGC GAG AAG AAA GTG GAC AGG	60	160
	CGT GGG CGC AAG GGT TA		
***JAG1***	TGC CGT TGC AGA AGT AAG AGT T	60	134
	TCC GCA GGC ACC AGT AGA A		
***JAG2***	TCG TCG TCA TCC CCT TCC A	60	107
	CTC GAT CAG CAG CTC CTC ATT C		
***HEY1***	GCA TAC GGC AGG AGG GAA A	60	181
	CTG GGA AGC GTA GTT GTT GAG AT		
***HEY2***	GCG TCG GGA TCG GAT AAA TAA	60	176
	CAA GAG CGT GTG CGT CAA AGT A		
***ACTB***	CGG CAT CGT CAC CAA CTG	60	70
	GGC ACA CGC AGC TCA TTG		

A 2-fold increased (a value ≥2) or decreased (a value ≤0.5) expression was considered biologically significant (overexpression or downregulation respectively). In each PCR reaction two negative controls were included, one with no cDNA template and one with no reverse transcription treatment. All qRT-PCR measurements were conducted in triplicates.

PCR products were electrophorized on 2% (w/v) agarose gels and stained with ethidium bromide, in order to verify that the corresponding product band (as visualized with the help of a UV transilluminator) had the correct size. Representative bands of all PCR products were extracted from the agarose gels, purified and sequenced, as a final confirmation step that the appropriate gene was being amplified at each PCR reaction.

### Protein extraction and Western blot (WB)

Human placental samples were homogenized at 4°C with 300μl lysis buffer (50mM Tris-HCl pH 7.4, 5mM EDTA, 250mM NaCl, 50mM NaF, 0.1% Triton X-100, 0.1mM Na_3_VO_4_, 1mM phenylmethane sulfonylfluoride, 10mg/ml leupeptin). Lysates were centrifuged at 14,000g for 10min at 4°C, and the supernatant was stored at -80°C until use. Protein levels were determined with the Bradford assay and normalized by Coomassie Blue staining. Equal amounts of total protein (30μg) were resolved on 8% polyacrylamide Tris/glycine gels and transferred onto nitrocellulose membranes in 10mM CAPS pH 11, which contained 10% methanol. Membranes were blocked for 1h at room temperature with PBS containing 0.1% Tween 20 (PBS-T) and 5% (w/v) low-fat milk powder, and were subsequently incubated overnight at 4°C with the rabbit primary antibodies against NOTCH2 (NICD2) or NOTCH3 (NICD3) intracellular domains (Cell Signaling, Danvers, MA), both diluted 1:500 in PBS containing 0.1% Tween 20 (PBS-T) and 5% (w/v) BSA (Sigma-Aldrich, St. Louis, MO). After 1h incubation with the peroxidase-conjugated secondary goat anti-rabbit antibody (1:1000, in PBS-T with 1% low-fat milk, Cell Signaling), immune complexes were detected with the SuperSignal West Pico chemiluminescent substrate (Pierce, Rockford, IL), and were imprinted on X-ray films (Fujifilm, Tokyo, Japan). The mouse anti-Actin antibody (MAB 1501, Chemicon, Temecula, CA) was used in order to normalize NICD2 and NICD3 expression. Films were scanned and the protein lanes were quantified using the Photoshop CS2 image analysis software (Adobe Systems Inc., San Francisco, CA).

### Statistical analysis

*NOTCH1*, *NOTCH2*, *NOTCH3*, *NOTCH4*, *DLL1*, *DLL3*, *DLL4*, *JAG1*, *JAG2*, *HEY1* and *HEY2* mRNA levels, as well as NICD2 and NICD3 protein levels, were first evaluated by the one-sample Kolmogorov-Smirnov goodness-of-fit test, in order to determine whether they followed a normal distribution pattern. Depending on the results, Pearson’s or the non-parametric Spearman’s rank test was used in order to examine their relationship pair-wise and their association with continuous variables (maternal age, BMI, weight gain, gestational age at delivery, birth weight). Moreover, their association with categorical data (smoking habit, mode of delivery, child gender, parity) was examined using Student’s T test (after an assessment of the equality of variances using Levene’s test), or its non-parametric equivalents Mann-Whitney U and Kruskal-Wallis H tests. Additionally, the Chi-square (χ^2^) test, replaced by Fisher’s exact test when indicated by the analysis, was used to examine the studied genes’ expression status with the various clinicopathological parameters after stratification. Finally, univariate analysis, with gestation age, mode of delivery and smoking during pregnancy as co-factors, was used in order to correct the results for these differences among the study groups. Statistical analyses were 2-sided and were performed with the SPSS 11.5 software (SPSS, Chicago, IL). Statistical significance was set at the 95% level (p-value<0.05).

## Results

### Clinical Data

Baseline demographic characteristics and medical history information (maternal weight, height, age, parity, smoking, mode of delivery, fetal gender, birth weight and its calculated centiles) were recorded for all study subjects (Table 2). Women with PE gained less weight during their pregnancies than women with normal pregnancies (p = 0.015). Gestation age was also shorter by two weeks (p = 0.012) and babies born from women with PE weighted 800g less that than those born from women with normal pregnancies (p<0.001). Additionally, due to higher pregnancy risks, women with PE were more often subjected to caesarean section than women with uncomplicated pregnancies (p = 0.025). Finally, women that developed PE during pregnancy were more often smokers prior to conceiving than women that had normal pregnancies (p = 0.001).

**Table 2:** Clinical characteristics of the study groups.

	Preeclampsia-complicated pregnancies (n = 20)	Normal pregnancies (n = 20)	*P*-value
**Maternal age** (mean±SD, years)	29.0±4.7	29.9±4.6	0.52^a^
**BMI** (mean±SD)	26.1±8.1	22.3±2.5	0.12^b^
**Maternal weight gain** (mean±SD, Kg)	9.0±6.4	14.8±7.9	**0.015** ^**a**^
**Gestational age at delivery** (mean±SD, wks)	36.5±3.2	38.8±2.0	**0.012** ^**b**^
**Birth weight** (mean±SD, gr)	2315±674	3150±498	**<0.001** ^**a**^
**Birth weight centile** (mean±SD, %)	15.6±25.6	42.7±22.3	**0.001** ^**a**^
**Mode of delivery**			
Vaginal (%)	8 (40.0)	15 (75.0)	**0.025** ^**c**^
Elective Caesarean section (%)	12 (60.0)	5 (25.0)	
**Parity**			
Nulliparous (%)	13 (65.0)	11 (55.0)	0.52^c^
Multiparous (%)	7 (35.0)	9 (45.0)	
**Child gender**			
Male (%)	4 (20.0)	7 (35.0)	0.29^c^
Female (%)	16 (80.0)	13 (65.0)	
**Small for Gestation Age babies**			
Yes (%)	13 (65.0)	2 (10.0)	**<0.001** ^**c**^
No (%)	7 (35.0)	18 (90.0)	
**Smoking**			
Yes (%)	13 (65.0)	3 (15.0)	**0.001** ^**c**^
No (%)	7 (35.0)	17 (85.0)	
**Smoking during pregnancy**			
Yes (%)	4 (20.0)	1 (5.0)	0.34^d^
No (%)	16 (80.0)	19 (95.0)	

^a^ Student’s T test

^b^ Mann-Whitney U test

^c^ Chi-square test

^d^ Fisher’s exact test

All tests were 2-tailed.

### mRNA expression profiling

**Fig 1:**
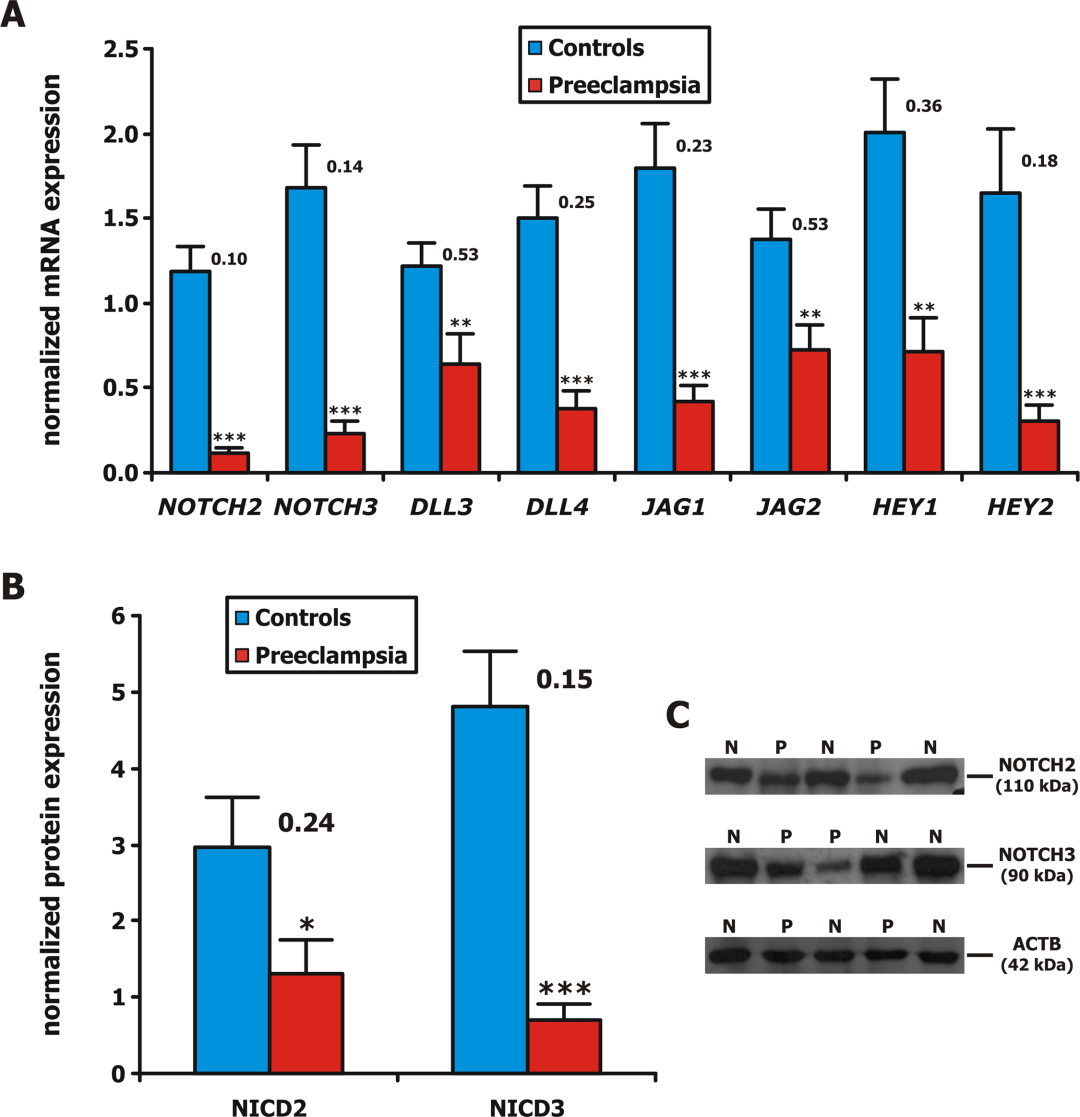
Bar chart depicting Notch pathway members normalized (A) mRNA and (B) protein expression in normal and preeclamptic (PE) late preterm and term placentas, respectively. Floating numbers represent fold change between the two sample groups. Asterisks represent statistically significant associations between PE and controls (*: p<0.05; **: p<0.01; ***: p<0.001). Error bars depict standard error of the mean (SEM). (C) Representative Western blots of NICD2 and NICD3 proteins, along with β-Actin (ACTB) housekeeping gene (N: Controls; P: Preeclampsia).

Our results revealed that *NOTCH1*, *NOTCH4*, and *DLL1* were not expressed in the late preterm or term placentas of either women with normal pregnancies or women with pregnancies complicated by PE. On the contrary, receptors *NOTCH2* and *NOTCH3*, ligands *DLL3*, *DLL4*, *JAG1* and *JAG2* and target genes *HEY1* and *HEY2* had decreased mRNA levels in PE samples, with an average expression ranging from 10% to 53% of the same gene’s expression in normal placentas (p<0.01 in all cases) (Table 3, Fig 1).

**Table 3:** Expression analysis of Notch signaling pathway between normal and PE placentas.

Gene	Preeclampsia Placentas	Normal Placentas	P-value (c)	P-value (a)	fold-change
**mRNA**					
***NOTCH2***	0.12 ± 0.03	1.18 ± 0.15	**<0.001**	**<0.001**	**0.10**
***NOTCH3***	0.23 ± 0.07	1.68 ± 0.25	**<0.001**	**<0.001**	**0.14**
***DLL3***	0.64 ± 0.17	1.22 ± 0.13	**0.002**	**0.027**	**0.53**
***DLL4***	0.38 ± 0.11	1.51 ± 0.19	**<0.001**	**0.001**	**0.25**
***JAG1***	0.42 ± 0.10	1.80 ± 0.26	**<0.001**	**<0.001**	**0.23**
***JAG2***	0.73 ± 0.14	1.38 ± 0.17	**0.008**	**0.024**	**0.53**
***HEY1***	0.72 ± 0.20	2.01 ± 0.31	**0.001**	**0.013**	**0.36**
***HEY2***	0.31 ± 0.09	1.65 ± 0.37	**<0.001**	**0.009**	**0.18**
**Protein**					
**NICD2**	1.31 ± 0.44	2.96 ± 0.66	**0.014**	**0.048**	**0.24**
**NICD3**	0.70 ± 0.20	4.81 ± 0.71	**<0.001**	**0.002**	**0.15**

**P-value (c):** crude p-value; **P-value (a):** P-value adjusted for gestation period, mode of delivery and smoking during pregnancy. All values are presented as mean ± SEM.

**Fig 2:**
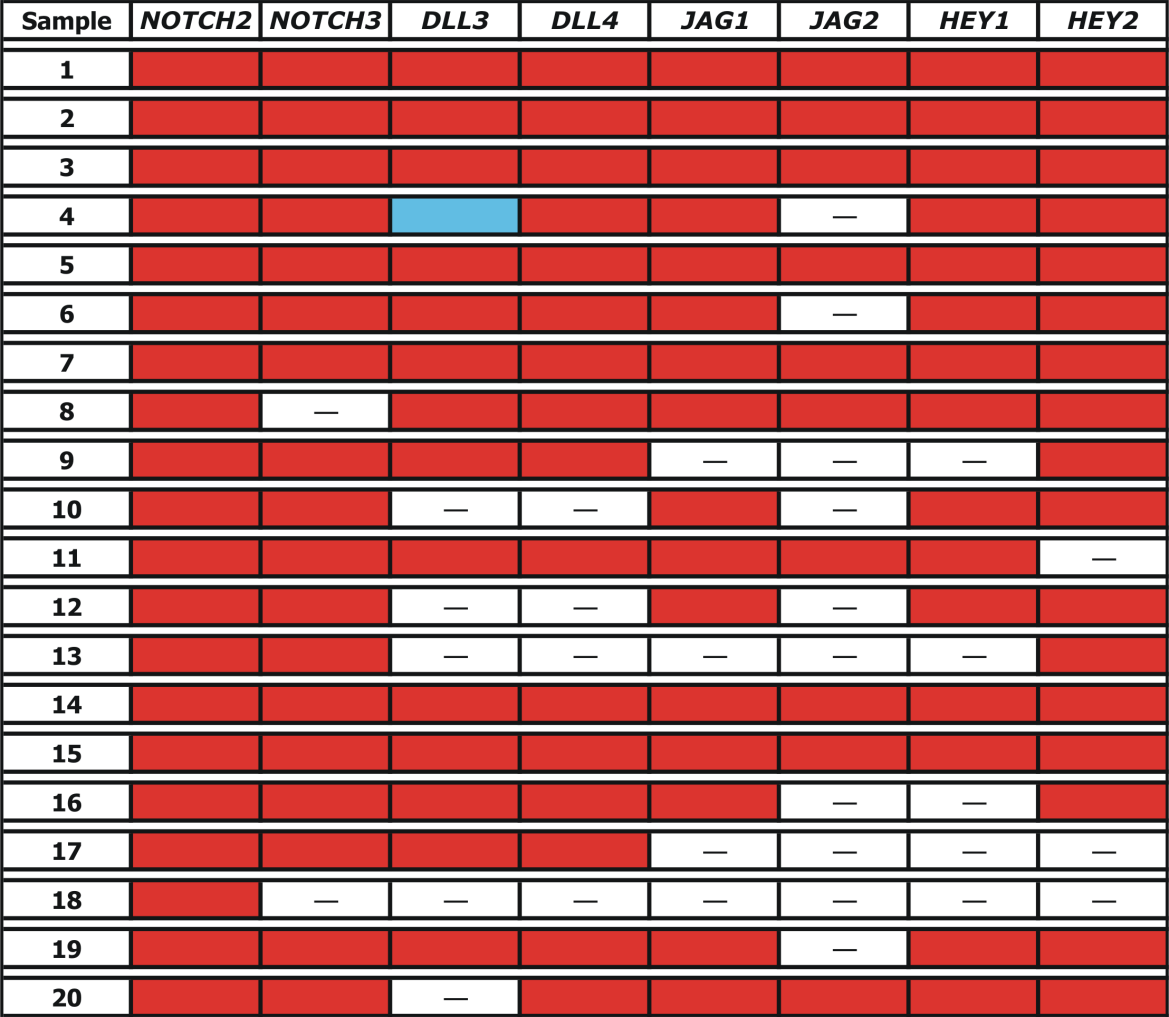
Schematic representation of Notch pathway molecules mRNA expression profile in our series of preeclampsia-complicated late preterm and term placentas. Red: Downregulation; White: Normal expression; Sky Blue: Overexpression.

However, the studied genes were not down-regulated in all PE samples (Fig 2). Compared to controls, the mRNA expression levels of *NOTCH2* and *NOTCH3* were downregulated in 100% (20/20) and 90% (18/20) of PE samples, respectively. Similarly, *DLL3*, *JAG1*, *JAG2*, *HEY1* and *HEY2* mRNA expression exhibited downregulation in 70% (14/20), 80% (16/20), 50% (10/20), 75% (15/20) and 85% (17/20) of PE samples, respectively.

### Western Blot measurements

Subsequently, since *NOTCH1* and *NOTCH4* were not expressed, the protein expression of the intracellular domains of only NOTCH2 (NICD2) and NOTCH3 (NICD3) receptors were measured by WB. As expected, we detected a decrease in the expression of NICD2 and NICD3 in PE samples compared with controls. The average expression of NICD2 and NICD3 in PE specimens was at 24% and 15% of the same protein’s expression in normal samples (p = 0.014 and p<0.001, respectively) (Table 3 & Fig 1). In accordance to the mRNA data, NICD2 was downregulated in 70% (14/20) of PE samples, while NICD3 was universally downregulated in 100% (20/20) of PE specimens.

### Statistical analysis

#### Univariate analysis

Univariate analysis of the data, using gestation age, mode of delivery and smoking during pregnancy as co-founding factors, revealed that the observed statistical differences in *NOTCH2*, *NOTCH3*, *DLL3*, *DLL4*, *JAG1*, *JAG2*, *HEY1*, *HEY2*, NICD2 and NICD3 levels between normal and PE-complicated placentas remained, even after correcting the data for possible discrepancies caused by the differences in the gestation period, delivery method and smoking status during pregnancy between the two groups (Table 3).

#### Associations with clinicopathological parameters

**Fig 3:**
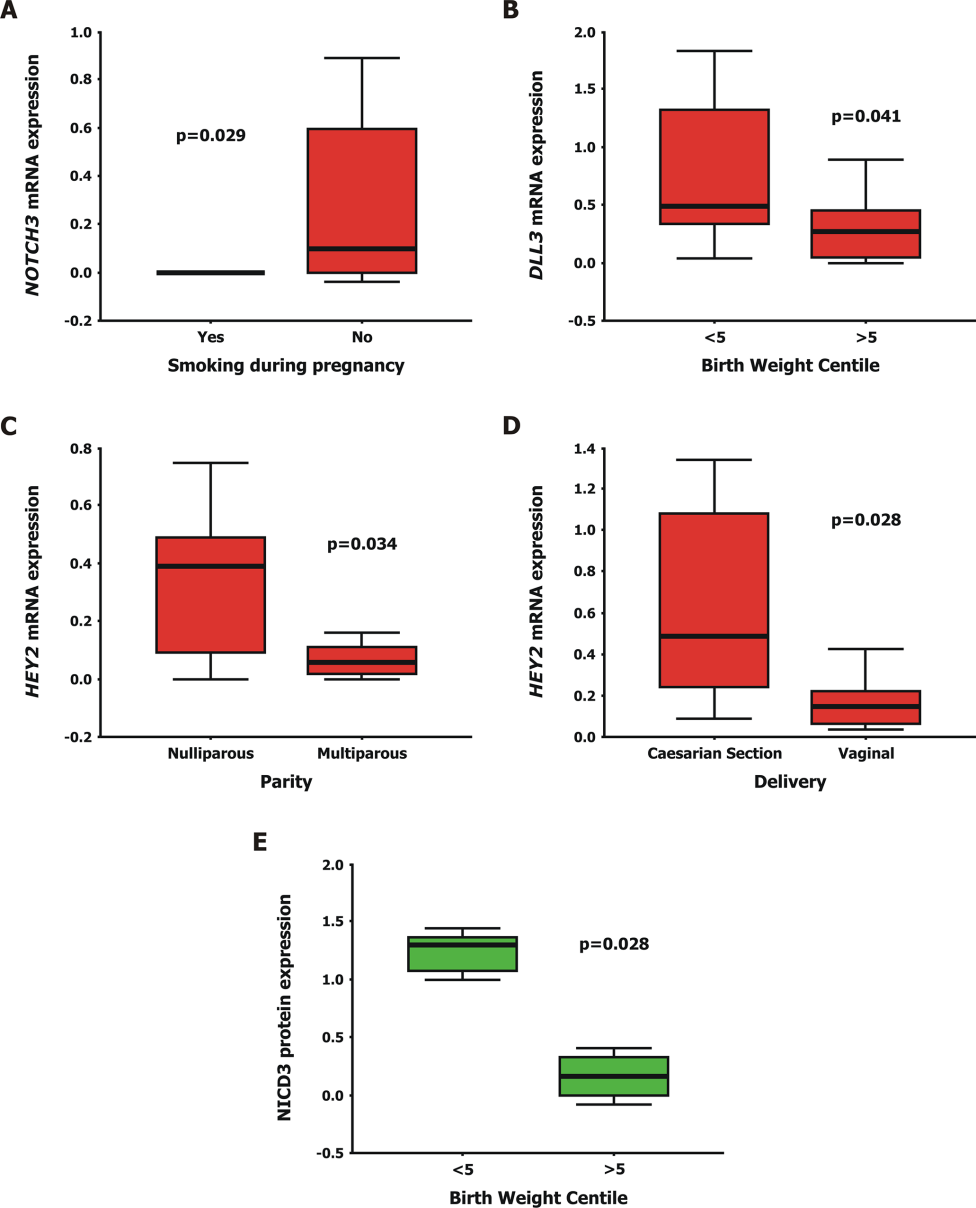
Box and whisker plots depicting statistically significant associations in preeclampsia-complicated late preterm and term placentas. (A) *NOTCH3* was not expressed in preeclamptic (PE) women that smoked during their pregnancy versus women with PE that did not smoke (0.00±0.00 vs. 0.29±0.09, p = 0.029). (B) *DLL3* mRNA expression was higher in babies born from PE pregnancies with Birth Weight Centile (BWC) <5 compared with babies born from PE pregnancies with BWC >5 (0.91±0.30 vs. 0.42±0.19, p = 0.041). (C) *HEY2* transcript levels were increased in women with PE who were on their fist parity versus women with PE that gave birth to their 2^nd^ or 3^rd^ child (0.44±0.13 vs. 0.05±0.02, p = 0.034). (D) *HEY2* mRNA expression was higher in women with PE who gave birth with a Caesarian Section compared with women with PE who gave birth naturally (0.63±0.17 vs. 0.15±0.05, p = 0.028). (E) NOTCH3 intracellular domain (NICD3) protein levels were higher in babies born from women with pregnancies complicated by PE with BWC <5 versus babies born from women with pregnancies complicated by PE with BWC >5 (1.15±0.24 vs. 0.16±0.07, p = 0.028). The thick line near the center of each rectangular box represents the median value, the bottom and top edges of the box indicate the 1^st^ (Q_1_) and 3^rd^ (Q_3_) quartiles, and the ends of the whiskers depict the 10^th^ (P_10_) and 90^th^ (P_90_) percentiles.

Statistical analysis revealed significant associations between the placental mRNA and protein levels of the studied molecules and certain pathological parameters in cases with PE (Fig 3). *NOTCH3* receptor was not expressed in women with PE that smoked during gestation compared with women with PE who did not smoke (0.00±0.00 versus 0.29±0.09, p = 0.029). Additionally, in babies born from women with PE with birth weight centile (BWC) <5 NICD3 protein levels and *DLL3* mRNA levels were higher compared with babies born from women with PE with BWC>5 (NICD3: 1.15±0.24 vs. 0.16±0.07, p = 0.028; *DLL3*: 0.91±0.30 vs. 0.42±0.19, p = 0.041). Finally, *HEY2* mRNA levels were increased in women with pregnancies complicated by PE for which an elective cesarean section was performed compared with those with a vaginal delivery (0.63±0.17 vs. 0.15±0.05, p = 0.028), and in women with PE who were on their first parity in comparison with women with PE that gave birth to their 2^nd^ or 3^rd^ child (0.44±0.13 vs. 0.05±0.02, p = 0.034).

#### Co-expression analysis

Using Spearman’s rank test, we tested the mRNA co-expression pattern of the eight expressed genes in a pair-wise manner in both PE and control samples (after normalization). This test examines whether two molecules are upregulated or downregulated together (positive association), or whether when one is overexpressed and the other has a reduced expression (negative association).

In term placentas from women with normal pregnancies we observed 12 co-expression pairs. *NOTCH3* was positively associated with *NOTCH2*. DLL4 was positively associated with receptors *NOTCH2*, *NOTCH3* and ligands *JAG1*, *JAG2* and *DLL3*. *JAG1* was positively associated with *NOTCH3*. *JAG2* was positively associated with *NOTCH3* and *JAG1*. *HEY1* target gene was positively associated with ligands *JAG1*, *JAG2* and *DLL4*. *HEY2* was not co-expressed with any of the other molecules (Table 4).

**Table 4:** Notch signaling pathway pair-wise mRNA co-expression analysis in normal placentas.

		*NOTCH2*	*NOTCH3*	*DLL3*	*DLL4*	*JAG1*	*JAG2*	*HEY1*	*HEY2*
***NOTCH2***	CC	1.000							
	P-value	–							
***NOTCH3***	CC	**0.767**	1.000						
	P-value	**<0.001**	–						
***DLL3***	CC	0.411	0.341	1.000					
	P-value	0.072	0.141	–					
***DLL4***	CC	**0.590**	**0.672**	**0.473**	1.000				
	P-value	**0.006**	**0.001**	**0.035**	–				
***JAG1***	CC	0.136	**0.521**	0.375	**0.519**	1.000			
	P-value	0.569	**0.018**	0.103	**0.019**	–			
***JAG2***	CC	0.147	**0.468**	0.357	**0.460**	**0.767**	1.000		
	P-value	0.535	**0.038**	0.123	**0.041**	**<0.001**	–		
***HEY1***	CC	0.120	0.417	0.253	**0.462**	**0.819**	**0.672**	1.000	
	P-value	0.613	0.068	0.282	**0.040**	**<0.001**	**0.001**	–	
***HEY2***	CC	0.050	0.337	-0.060	0.364	0.300	0.413	0.289	1.000
	P-value	0.835	0.146	0.801	0.115	0.199	0.070	0.217	–

**CC**: Correlation coefficient.

In late preterm and term placentas from women with PE we observed more (19 instead of 12) and different co-expressions between the studied genes than in normal samples. Specifically, the positive associations of *NOTCH3* with *NOTCH2* and *DLL4* with *JAG1* were lost in PE samples. Additionally, both *JAG1* and *JAG2* were not co-expressed with *NOTCH3*, but with *NOTCH2* instead. *HEY1* was additionally associated with *NOTCH2* and *DLL3*, and *HEY2* was co-expressed with the other 7 molecules (*NOTCH2*,*-3*, *JAG1*,*-2*, *DLL3*,*-4* and *HEY1*) (Table 5).

**Table 5:** Notch signaling pathway pair-wise mRNA co-expression analysis in preeclamptic placentas.

		*NOTCH2*	*NOTCH3*	*DLL3*	*DLL4*	*JAG1*	*JAG2*	*HEY1*	*HEY2*
***NOTCH2***	CC	1.000							
	P-value	–							
***NOTCH3***	CC	0.412	1.000						
	P-value	0.071	–						
***DLL3***	CC	0.264	0.128	1.000					
	P-value	0.261	0.591	–					
***DLL4***	CC	**0.789**	**0.628**	0.215	1.000				
	P-value	**<0.001**	**0.003**	0.362	–				
***JAG1***	CC	**0.877**	0.364	0.341	**0.766**	1.000			
	P-value	**<0.001**	0.115	0.141	**<0.001**	–			
***JAG2***	CC	**0.807**	0.269	0.401	**0.715**	**0.922**	1.000		
	P-value	**<0.001**	0.252	0.080	**<0.001**	**<0.001**	–		
***HEY1***	CC	**0.799**	0.400	**0.504**	**0.700**	**0.894**	**0.890**	1.000	
	P-value	**<0.001**	0.080	**0.024**	**0.001**	**<0.001**	**<0.001**	–	
***HEY2***	CC	**0.693**	**0.514**	**0.502**	**0.548**	**0.709**	**0.616**	**0.741**	1.000
	P-value	**0.001**	**0.020**	**0.024**	**0.012**	**<0.001**	**0.004**	**<0.001**	–

**CC**: Correlation coefficient.

## Discussion

In the present study, we found that receptors *NOTCH2* and *NOTCH3* and their ligands *DLL3*, *DLL4*, *JAG1* and *JAG2* had reduced mRNA expression in PE placentas compared to controls. Deficiencies in *NOTCH1*, *NOTCH2*, *JAG1* and *DLL4* result in the failed incorporation of the placental arterial vasculature into the maternal circulation [14]. Additionally, JAG1, along with VEGF, are significantly decreased in PE placentas, a finding believed to play a role in the onset of this pregnancy complication [15]. Other studies, however, have failed to detect any differences in *NOTCH2* and *DLL4* expression between PE and normal placentas [8], have observed an increase in JAG1 expression through pregnancy [16], or while agreeing with our observations regarding NOTCH2 downregulation, they found NOTCH3 to be overexpressed [17], albeit in early-onset severe preeclampsia (gestation period 24–33 wks).

Receptors *NOTCH1* and *NOTCH4* and the ligand *DLL1* were not expressed in the placenta of both normal and PE-complicated pregnancies. This is in accordance with a previous study in which *NOTCH1* was not expressed in CTBs and *NOTCH4* levels were noticeably reduced in extravillous trophoblast cells [8]. However, other reports suggest that both NOTCH1 and NOTCH4 are expressed in the placenta [6, 15, 16]. Possible explanations regarding expression variations between our study and previous ones are the different experimental procedures used, since we measured expression with qRT-PCR while those studies used either Western Blot or immunohistochemistry, the different ethnic background of the study populations, and the temporal and spatial heterogeneity of the placental structures, since it is believed that CTBs alter the expression of Notch receptors and ligands as they differentiate.

NICD2 and NICD3 protein levels were also lower in PE placentas versus normal ones, a finding in accordance with the reduced mRNA levels of *NOTCH2* and *NOTCH3* in our PE cohort. A significant decrease in the immunoreactivity of NOTCH2 and JAG2 proteins has been observed before in human term placentas complicated by fetal growth restriction (FGR) or hypertension, compared to normal placentas [18].

Furthermore, our findings revealed that the Notch target genes *HEY1* and *HEY2* had a decreased mRNA expression in PE placentas compared with controls. Although no previous data on *HEY* genes expression in PE exist, their reduced mRNA levels are probably a direct result of the universal downregulation of the Notch signal transduction mechanism. This can be verified by our co-expression analysis, especially for *HEY2*, which was co-downregulated with *NOTCH2*, *NOTCH3*, *JAG1*, *JAG2*, *DLL3*, *DLL4* and *HEY1* in PE placentas, while it was not co-expressed with any of the aforementioned genes in normal placentas. However, the different interaction of *HEY2* with the other Notch pathway molecules in PE-complicated placentas versus controls could also indicate that it plays a significant role in the development and progression of PE, a finding which warrants further investigation.

According to our results, some of the studied genes in the PE and control samples were positively associated with each other. However, certain co-expressions were lost, while others were gained in placentas from women with PE versus control placentas. Additionally, in PE cases a greater number of co-expressions (19 instead of 12) were observed compared to those in normal samples. The diverse interplay between these molecules in PE-affected placentas compared to normal ones could be a key event in PE pathogenesis.

Statistical analysis revealed that PE-affected pregnancies with BWC<5 had increased *DLL3* mRNA and NICD3 protein levels compared with PE-affected pregnancies with BWC>5. This observation is similar to one in our previous study on pregnancies complicated by FGR, in which *PHD3*, a member of the HIF hypoxia pathway, was higher in babies born from FGR pregnancies with BWC<0.5 compared to babies born from FGR pregnancies with BWC>0.5 [19]. Additionally, in women with PE that were on their first parity, *HEY2* transcript levels were increased compared with PE women that gave birth to their 2^nd^ or 3^rd^ child. Several studies suggest that gene expression alters between the 1^st^, 2^nd^ and 3^rd^ pregnancy, as in the case of sFLT1, whose levels are higher in the 1^st^ pregnancy compared with its levels in subsequent ones [20]. Moreover, Hey-2 mRNA expression was higher in women with PE that gave birth with a cesarean section compared with women with PE that gave birth naturally, a finding also observed for *PHD3* in our FGR study [19], and can probably be attributed to less stress during delivery and the fact that women that gave birth with a cesarean section had more severe preeclampsia than women who gave birth naturally. Finally, in women with PE that smoked during their gestation, receptor *NOTCH3* was not expressed, while in women with PE that did not smoke *NOTCH3* was expressed. Tobacco smoking downregulates the expression of Notch pathway members in the lung epithelium, both in smokers and chronic obstructive pulmonary disease (COPD) patients [21], and causes maternal endothelial dysfunction and abnormal placentation [22], a finding consistent with our results.

The main strength of our research is that it is the first study to concurrently measure the mRNA levels of all key Notch signaling pathway molecules, and especially target genes *HEY1* and *HEY2*, whose expression in human placenta have never been studied before, and to determine the associations between them. Study limitations include the fact that our research was conducted only on late preterm and term placentas, and therefore we were not able to measure the expression of the Notch pathway molecules during the earlier stages of pregnancy, and that our samples were whole tissue homogenates and not micro-dissected, which means that the measured expression of the Notch signaling pathway genes was their average expression among the various placenta cell types and not their expression in each cell type individually. Evidently, these limitations could also explain the differences between our results and those of some previously conducted studies. Another potential limitation would be the differences in gestation period (shorter in PE cases), mode of delivery (since elective caesarean sections were more common in PE samples than in controls) and smoking status (since more women with PE smoked) between the two groups, which could introduce a possible bias in the results. However, since univariate analysis, with the aforementioned parameters as co-factors, found that the differences in the expression of all studied genes between PE cases and controls retained their statistical significance, our findings cannot be attributed to sampling bias, but are linked to PE pathophysiology.

## Conclusions

**Fig 4:**
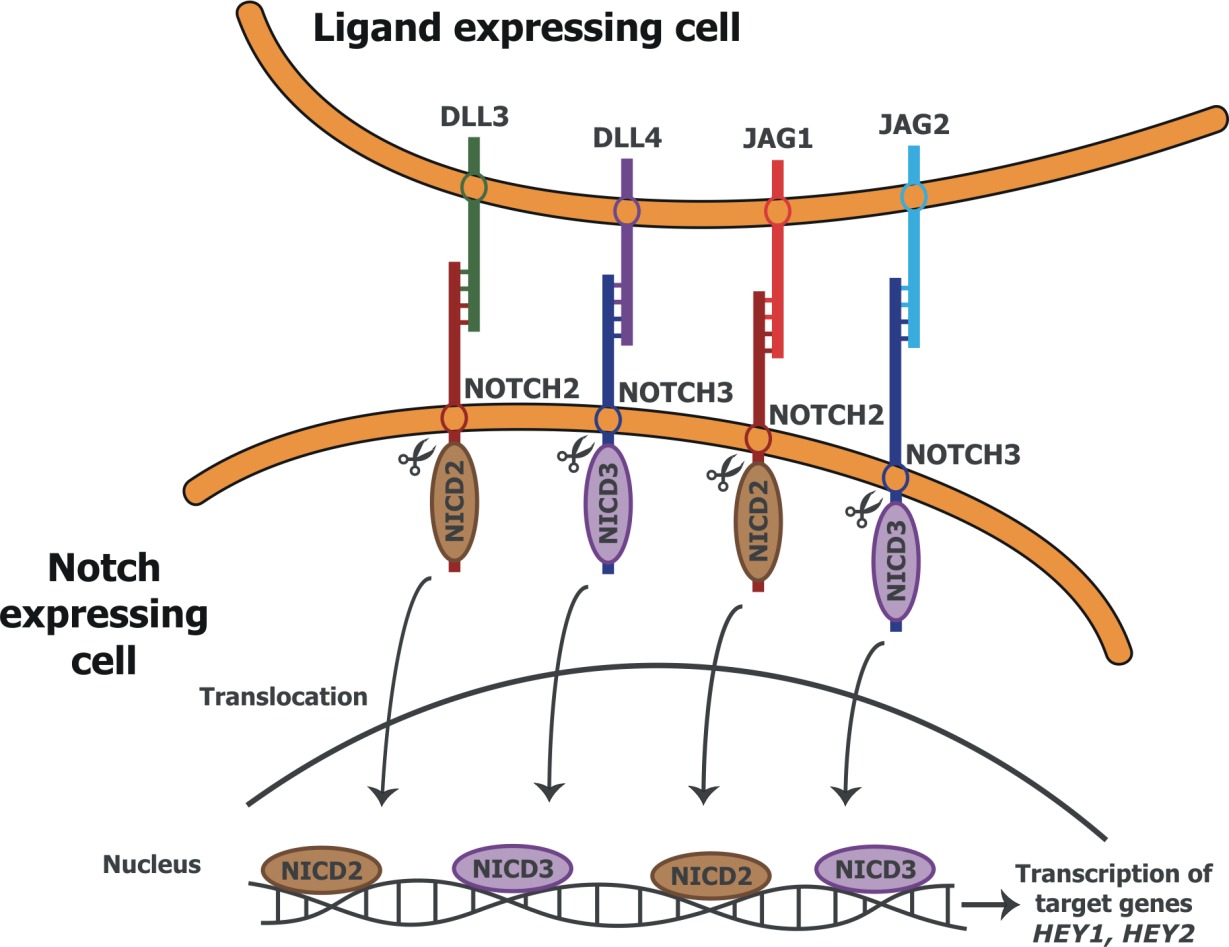
Schematic depiction of the studied Notch signaling pathway molecules in late preterm and term placentas.

Our findings, that receptors *NOTCH2*,*-3*, ligands *DLL3*,*-4* and *JAG1*,*-2* and target genes *HEY1*,*-2* are downregulated in PE-complicated late preterm and term placentas, provide evidence that the Notch signaling pathway is associated with this pregnancy complication (visual depiction of the pathway in Fig 4). Further research regarding the function of Notch receptors, ligands and target genes in the placenta is required in order to elucidate their role in PE pathogenesis and to determine whether one or more of these genes could be used as biomarkers for the early detection of PE, or for new preventive and therapeutic strategies.

## References

[1] Roberts JM, Cooper DW. Pathogenesis and genetics of pre-eclampsia. Lancet. 2001; 357: 53–56. 11197372 10.1016/s0140-6736(00)03577-7

[2] Young BC, Levine RJ, Karumanchi SA. Pathogenesis of preeclampsia. Annu Rev Pathol. 2010; 5: 173–192. 10.1146/annurev-pathol-121808-102149 20078220

[3] Artavanis-Tsakonas S, Rand MD, Lake RJ. Notch signaling: cell fate control and signal integration in development. Science. 1999; 284: 770–776. 10221902 10.1126/science.284.5415.770

[4] Fleming RJ. Structural conservation of Notch receptors and ligands. Semin Cell Dev Biol. 1998; 9: 599–607. 9918871 10.1006/scdb.1998.0260

[5] Borggrefe T, Oswald F. The Notch signaling pathway: transcriptional regulation at Notch target genes. Cell Mol Life Sci. 2009; 66: 1631–1646. 10.1007/s00018-009-8668-7 19165418 PMC11115614

[6] Herr F, Schreiner I, Baal N, Pfarrer C, Zygmunt M. Expression patterns of Notch receptors and their ligands Jagged and Delta in human placenta. Placenta. 2011; 32: 554–563. 10.1016/j.placenta.2011.04.018 21726900

[7] Gasperowicz M, Otto F. The notch signalling pathway in the development of the mouse placenta. Placenta. 2008; 29: 651–659. 10.1016/j.placenta.2008.06.004 18603295

[8] Hunkapiller NM, Gasperowicz M, Kapidzic M, Plaks V, Maltepe E, Kitajewski J, et al A role for Notch signaling in trophoblast endovascular invasion and in the pathogenesis of pre-eclampsia. Development. 2011; 138: 2987–2998. 10.1242/dev.066589 21693515 PMC3119307

[9] Fischer A, Schumacher N, Maier M, Sendtner M, Gessler M. The Notch target genes Hey1 and Hey2 are required for embryonic vascular development. Genes Dev. 2004; 18: 901–911. 15107403 10.1101/gad.291004 PMC395849

[10] High FA, Epstein JA. The multifaceted role of Notch in cardiac development and disease. Nat Rev Genet. 2008; 9: 49–61. 18071321 10.1038/nrg2279

[11] Kume T. Ligand-dependent Notch signaling in vascular formation. Adv Exp Med Biol. 2012; 727: 210–222. 10.1007/978-1-4614-0899-4_16 22399350

[12] Zhao WX, Lin JH. Notch signaling pathway and human placenta. Int J Med Sci. 2012; 9: 447–452. 10.7150/ijms.4593 22859905 PMC3410364

[13] ACOG Committee on Practice Bulletins—Obstetrics. ACOG practice bulletin. Diagnosis and management of preeclampsia and eclampsia. Number 33, January 2002. Obstet Gynecol. 2002; 99: 159–167. 16175681 10.1016/s0029-7844(01)01747-1

[14] Ji L, Brkic J, Liu M, Fu G, Peng C, Wang YL. Placental trophoblast cell differentiation: physiological regulation and pathological relevance to preeclampsia. Mol Aspects Med. 2013; 34: 981–1023. 10.1016/j.mam.2012.12.008 23276825

[15] Cobellis L, Mastrogiacomo A, Federico E, Schettino MT, De Falco M, Manente L, et al Distribution of Notch protein members in normal and preeclampsia-complicated placentas. Cell Tissue Res. 2007; 330: 527–534. 17955263 10.1007/s00441-007-0511-6

[16] De Falco M, Cobellis L, Giraldi D, Mastrogiacomo A, Perna A, Colacurci N, et al Expression and distribution of notch protein members in human placenta throughout pregnancy. Placenta. 2007; 28: 118–126. 17185135 10.1016/j.placenta.2006.03.010

[17] Zhao WX, Huang TT, Jiang M, Feng R, Lin JH. Expression of notch family proteins in placentas from patients with early-onset severe preeclampsia. Reprod Sci. 2014; 21: 716–723. 10.1177/1933719113512530 24336671 PMC4016722

[18] Sahin Z, Acar N, Ozbey O, Ustunel I, Demir R. Distribution of Notch family proteins in intrauterine growth restriction and hypertension complicated human term placentas. Acta Histochem. 2011; 113: 270–276. 10.1016/j.acthis.2009.10.006 19913284

[19] Gourvas V, Sifakis S, Dalpa E, Soulitzis N, Koukoura O, Spandidos DA. Reduced placental prolyl hydroxylase 3 mRNA expression in pregnancies affected by fetal growth restriction. BJOG. 2010; 117: 1635–1642. 10.1111/j.1471-0528.2010.02735.x 21040392

[20] Wolf M, Shah A, Lam C, Martinez A, Smirnakis KV, Epstein FH, et al Circulating levels of the antiangiogenic marker sFLT-1 are increased in first versus second pregnancies. Am J Obstet Gynecol. 2005; 193: 16–22. 16021053 10.1016/j.ajog.2005.03.016

[21] Tilley AE, Harvey BG, Heguy A, Hackett NR, Wang R, O'Connor TP, et al Down-regulation of the notch pathway in human airway epithelium in association with smoking and chronic obstructive pulmonary disease. Am J Respir Crit Care Med. 2009; 179: 457–466. 10.1164/rccm.200705-795OC 19106307 PMC2654975

[22] Ness RB, Zhang J, Bass D, Klebanoff MA. Interactions between smoking and weight in pregnancies complicated by preeclampsia and small-for-gestational-age birth. Am J Epidemiol. 2008; 168: 427–433. 10.1093/aje/kwn140 18558661 PMC2562690

